# The Potential Prebiotic Berberine Combined With Methimazole Improved the Therapeutic Effect of Graves’ Disease Patients Through Regulating the Intestinal Microbiome

**DOI:** 10.3389/fimmu.2021.826067

**Published:** 2022-01-10

**Authors:** Zhe Han, Chaoping Cen, Qianying Ou, Yonggui Pan, Jiachao Zhang, Dongxue Huo, Kaining Chen

**Affiliations:** ^1^ School of Food Science and Engineering, Hainan University, Department of Endocrinology, Hainan General Hospital, Hainan Affiliated Hospital of Hainan Medical University, Haikou, China; ^2^ Key Laboratory of Food Nutrition and Functional Food of Hainan Province, Hainan University, Haikou, China

**Keywords:** berberine, methimazole, Graves’ disease, microbiome, prebiotics

## Abstract

Graves’ disease, a typical metabolism disorder, causes diffuse goiter accompanied by ocular abnormalities and ocular dysfunction. Although methimazole (MI) is a commonly used drug for the treatment of GD, the efficacy of methimazole is only limited to the control of clinical indicators, and the side effects of MI should be seriously considered. Here, we designed a 6-month clinical trial that divided the patients into two groups: a methimazole group (*n*=8) and a methimazole combined with potential prebiotic berberine group (*n*=10). The effects of both treatments on thyroid function and treatment outcomes in patients with GD were assessed by thyroid index measurements and gut microbiota metagenomic sequencing. The results showed that the addition of berberine restored the patients’ TSH and FT3 indices to normal levels, whereas MI alone restored only FT3. In addition, TRAb was closer to the healthy threshold at the end of treatment with the drug combination. MI alone failed to modulate the gut microbiota of the patients. However, the combination of berberine with methimazole significantly altered the microbiota structure of the patients, increasing the abundance of the beneficial bacteria *Lactococcus lactis* while decreasing the abundance of the pathogenic bacteria *Enterobacter hormaechei* and *Chryseobacterium indologenes*. Furthermore, further mechanistic exploration showed that the addition of berberine resulted in a significant upregulation of the synthesis of enterobactin, which may have increased iron functioning and thus restored thyroid function. In conclusion, methimazole combined with berberine has better efficacy in patients with GD, suggesting the potential benefit of berberine combined with methimazole in modulating the composition of intestinal microbes in the treatment of GD, providing new strong evidence for the effectiveness of combining Chinese and Western drugs from the perspective of modulating the intestinal microbiota.

## Introduction

Graves’ disease (GD), a manifestation of autoimmune thyroid disease (AITD), is caused by an immune attack on the thyroid caused by an imbalance in the immune system (1). According to recent epidemiological surveys, the annual incidence of GD ranges from 20 to 50 cases per 100,000 people (2), with a higher incidence in women than in men (a male to female ratio of approximately 1:6), and it primarily manifests in middle age (3, 4). Studies have shown that GD is induced by a combination of genetic, environmental and autoimmune factors (5–7). GD is characterized by an abnormal increase in the secretion of thyroid hormones and is commonly associated with diffuse goiter (8). The onset of the disease is usually accompanied by a variety of complications, such as ocular deformities and ocular dysfunction, which significantly reduce the quality of life of patients with GD (9).

Inhibition of thyroid hormone synthesis using thionamides (including imidazoles and thioureas) is the basic treatment for Graves’ hyperthyroidism in clinical practice (8, 10). Methimazole(MI), which belongs to the imidazole class, has become a first-line drug in clinical practice because it can act on multiple pathways, including internal thyroid and antibody regulation (11). However, methimazole can cause premature delivery and foetal malformations in pregnant patients (12). Additionally, methimazole is a risk factor for hypothyroidism in infants of mothers who are breastfeeding because of the presence of methimazole in their milk (13). In addition, prolonged use of methimazole in patients with GD can produce adverse drug reactions such as granulocyte deficiency, allergic rash and vasculitis (14). Although methimazole relieves the clinical signs and symptoms of hyperthyroidism, the cure rate is generally low (15). Therefore, its efficacy is limited to the control of clinical indicators (16) and its use is somewhat unsatisfactory in the treatment of GD.

At present, combinations of Chinese and Western medicines are increasingly being used in clinical practice. The combination of Chinese and Western medicine not only improves the clinical efficacy of treatments for type 2 cardiorenal syndrome and significantly enhances cardiorenal function (17) but it also outperforms conventional Western medicine alone in the treatment of AIDS (18), hyperlipidaemia (19)and type 2 diabetes (20) mellitus and it has a good safety profile. Chinese herbal medicine is often used as a powerful supplement to Western medicine, and the combination of Chinese and Western medicines can play a role in increasing the treatment effectiveness while reducing its toxicity (21).

Chinese herbal medicines play a complex role in the treatment of disease, and numerous studies have confirmed that the essence of the effectiveness of Chinese herbal medicines is the prebiotics of the intestinal microbiota. Many of the components of the beneficial tonic herbs such as ginseng and ganoderma lucidum have prebiotic effects, such as polysaccharides. Ginseng soluble dietary fibre (22) and Lycium barbarum polysaccharides (23) can be fermented by the intestinal microbiota to produce beneficial metabolites such as short-chain fatty acids, thus promoting the growth of beneficial bacteria (e.g. lactic acid bacteria) and displaying prebiotic properties. In fact, berberine also acts as a prebiotic in the intestinal tract. In the intestine berberine reduces inflammation by regulating the intestinal microbiota and promoting the production of butyrate by the strains of bacteria. In addition, the potential prebiotic berberine has shown powerful effects in the treatment of metabolic diseases such as hyperlipidemia (24) and diabetes (25). Graves’ disease, known as Yin deficiency and Yang hyperactivity in Chinese medicine, is clinically characterized by metabolic disorders such as hyperphagia, palpitations and night sweats. In traditional Chinese medicine, berberine is considered to be effective in clearing heat and dampness, dipping fire and detoxifying toxins, while in western medicine it is clinically useful in treating metabolic disorders, so it is feasible to apply berberine in the treatment of Graves’ disease.

**Figure 1:**
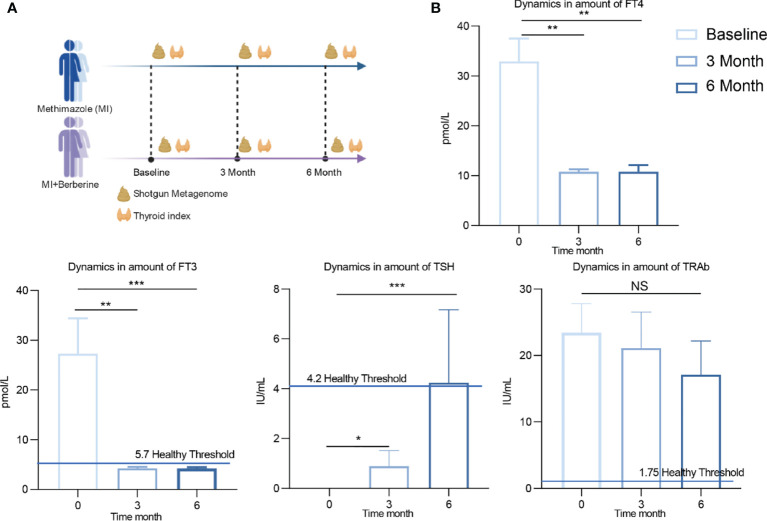
Experimental design and changes of clinical indexes after treatment with methimidazole. **(A)** The study was divided into two groups, the methimazole treatment group (*n*=8) and the combined methimazole and berberine treatment group (*n*=10) both groups were treated for 6 months. Serum and stool samples were collected at baseline, 3 months and 6 months for measurement of thyroid parameters and shotgun metagenomic sequencing, respectively. **(B)** Concentrations of thyroid indicators such as free triiodothyronine (FT3), free thyroxine (FT4), thyroid stimulating hormone (TSH) and thyroid stimulating hormone receptor antibody (TRAb) at baseline, 3 months of treatment and 6 months of treatment. The differences in the levels of thyroid indicators between the two groups were compared using the Kruskal-Wallis test, and the differences between the two groups were statistically significant. Asterisks indicate significant differences between baseline and endpoints in the corresponding groups (***P < 0.001, **P < 0.01, *P < 0.05) NS means there is no significant difference between the two groups, error line means ± SEM values.

Although there are many studies on the combination of Chinese and Western medicine in the treatment of diseases, few reports have analysed the mechanism of drug action in depth from the perspective of the intestinal microbiota. Therefore, to fill this gap, it is important to explore how the intestinal microbiota responds to drugs to help the host maintain internal environmental homeostasis and improve the treatment efficacy. We designed a 6-month clinical trial to divide patients with GD into a methimazole treatment group (traditional Western medicine) and a methimazole + berberine treatment group (combination of Western and Chinese medicines) (**Figure 1A**). During the trial, the changes in thyroid indices were recorded at baseline and three months and six months during treatment, and the gut microbiota at different time points was determined by shotgun metagenome sequencing. Our study provides a preliminary exploration of the mechanism by which the combination of Chinese and Western drugs regulates the intestinal microbiota of patients with GD and improves their therapeutic effect, and it lays a foundation for an analysis of the combination of Chinese and Western drugs for the treatment of metabolic diseases based on the perspective of intestinal microbiology.

## Material and Methods

### Experimental Design and Volunteer Recruitment

All volunteers in our experiment were from Hainan Provincial People’s Hospital (Haikou, China). The experiment was divided into two groups: the methimazole treatment group (*n* = 8, Methimazole 20mg per tablet, 1 tablet each time once daily) and the combined methimazole and berberine treatment group (*n* = 10, Methimazole 20mg per tablet, 1 tablet each time once daily + Berberine tablets 0.1g per tablet, three tablets each time three times a day). All volunteers received treatment for a period of 6 months.

Our study was reviewed and approved by the Hainan Provincial People’s Hospital (2018–109), and the sampling and follow-up steps during the study were performed according to the approved guidelines.

We collected blood and stool from the volunteers at baseline and at 3 months and 6 months of treatment, which were performed by physicians while the patients were under clinical care in the hospital. We weighed the stool samples and added a protectant to the samples at a ratio of 1:5 to protect the nucleotides. All samples were stored at -20°C until subsequent processing.

### Measurement of Clinical Indicators

Free triiodothyronine (FT3) and free thyroxine (FT4), thyroid-stimulating hormone (TSH) and thyroid-stimulating hormone receptor antibodies (TRAb) were measured by enzyme-linked immunosorbent assay.

DNA was extracted from the stool samples using a Stool Mini Kit (Qiagen, Hilden, Germany) using Stool AMP ^®^ DNA. The DNA mass was calculated by 0.8% agarose electrophoresis, and DNA OD260/280 was measured by spectrophotometry. Novogene’s Illumina HiSeq 2500 instrument was used to perform shotgun metagenomic sequencing of all of the DNA samples. A DNA fragment of approximately 300 bp was used to build the library. We used 100 bp forwards and reverse to produce paired end readings. FastQC was used to control the quality of the readings, and then the data were compared with the human genome to remove the host genes.

### Identification of Microbial Species and Metabolic Pathways

MEGAHIT (26) was used to assemble the shotgun readings into contigs, scaffolds and mounts using the original parameters. Bracken software (27) was used to annotate the metagenomic species. Based on the UniRef90 database, we used HUMAN2 (28) to annotate the metagenomic functional features and metabolic pathways.

### Construction of the Metagenomic Assembled Genomes (MAGs)

We analysed the macrogenomic species by constructing MAGs, which were constructed using MetaBAT (29) for the binding of shotgun reads. After binning, the MAGs were assigned to a given reference genome if Prodigal identified more than 80% of the subgenes and more than 90% homology with the same genome using a BLASTn threshold of more than 95% for the same genome. Next, the classification annotations of MAGS were performed using GTDB-TK (V1.40) software (30). The parameters for applying this software for taxonomic assignment in this study were set with reference to Huo (31).

## Result

### Methimazole Intervention Improved Thyroid Function But Failed to Change Gut Microbes in Patients With GD

Methimazole restored thyroid function, significantly reduced FT3 and FT4 and significantly increased TSH by the end of treatment compared to baseline (**Figure 1B**). TSH is one of the hormones secreted by the anterior pituitary gland, and its main function is to control and regulate the activity of the thyroid gland. FT3 and FT4 are one of the most sensitive indicators for diagnosing hyperthyroidism. It is worth noting that the patient’s FT3 returned to healthy levels (5.7 pmol/L) after 6 months of methimazole treatment. TRAb is an important index that reflects the recovery of thyroid patients. Although TRAb decreased after treatment, the average level of TRAb still did not reach the normal range of healthy individuals (1.75 IU/mL).

**Figure 2:**
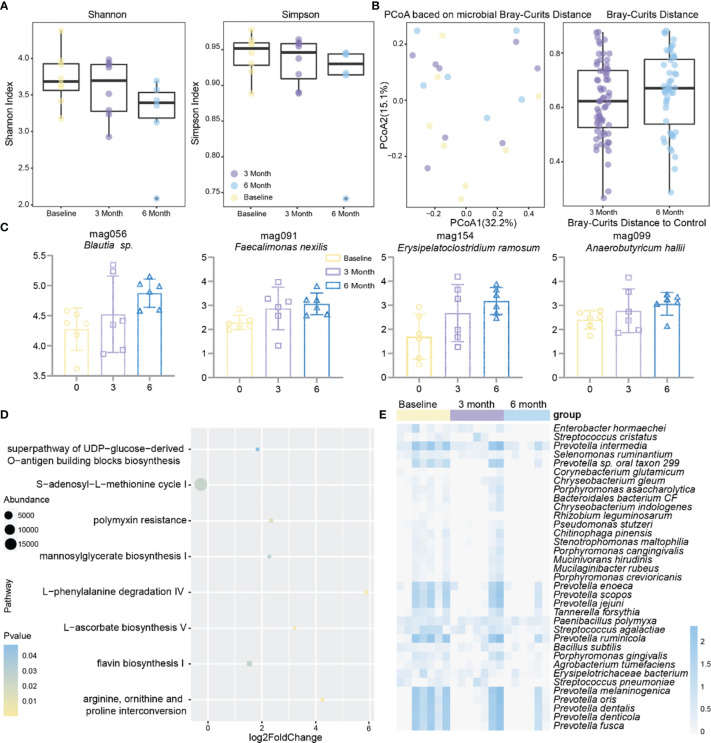
Effect of methimazole alone on the intestinal microbiota of patients with GD. **(A)** The effects of mebendazole alone on intestinal microbiota α diversity, including shannon and Simpson index, the same color points represent the volunteers at different time points. **(B)** Principal coordinates analysis (PCoA) based on Bray-Curtis distances for metagenomic species, with points of the same color representing the volunteer at different time points. **(C)** MAGs with significant differences from baseline at 6 months of treatment. **(D)** Gut microbial metabolic pathways that produced significant differences from methimazole treatment at 6 months of treatment at baseline. **(E)** Strains with significant differences from baseline at 6 months of treatment.

To investigate the effect of methimazole on patients’ intestinal microbes, stool samples were collected at baseline, at 3 months of treatment, and at 6 months of treatment, and changes in intestinal microbes during the treatment period were analysed by shotgun metagenomic sequencing. Although methimazole treatment did not significantly change the Shannon or Simpson indexes after 6 months, they both showed a decreasing trend (**Figure 2A**). Subsequently, we calculated the microbial Bray–Curtis distance for each subject from baseline to each time point (**Figure 2B**). Unfortunately, methimazole failed to alter the structure of the gut microbiota of the subjects. However, we sorted out the species that had significant changes at the three time points, in which the abundance of some species of *Prevotella* spp. decreased significantly. The abundance of some other strains, such as *Streptococcus pneumoniae*, *Selenomonas ruminantium*, and *Enterobacter hormaechei*, also decreased significantly (**Figure 2E**). The MAGs *Faecalimonas nexilis*, *Erysipelatoclostridium ramosum, Anaerobutyricum hallii* and *Blautia* sp. increased significantly at the three time points (**Figure 2C**).

While focusing on changes in the gut microbiota, we further investigated changes in the metabolic pathways in the subjects at baseline compared to other time points during treatment (**Figure 2D**). We found significant increases in metabolic pathways such as L-ascorbate biosynthesis V and L-phenylalanine degradation IV after six months of methimazole treatment.

The above results suggested that methimazole has a therapeutic effect in patients with GD. Although some species such as *Prevotella* spp. and *Streptococcus pneumoniae* were significantly reduced after drug administration, there was no effect on the species homogeneity and diversity of the gut microbiota in patients with GD.

### The Combination of Methimazole and the Potential Prebiotic Berberine Improves the Efficacy in Restoring Thyroid Function in Patients With GD and Significantly Alters the Intestinal Microbiota

**Figure 3:**
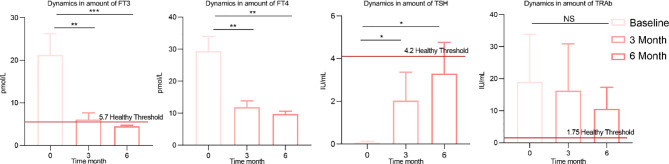
Changes in clinical indicators after berberine combined with methimazole treatment. The concentrations of thyroid indicators such as free triiodothyronine (FT3), free thyroxine (FT4), thyroid-stimulating hormone (TSH), and thyroid-stimulating hormone receptor antibodies (TRAb) at baseline, 3 months of treatment and 6 months of treatment. The differences in the levels of thyroid indicators between the two groups were compared using the Kruskal-Wallis test, and the differences between the two groups were statistically significant. Asterisks indicate significant differences between baseline and endpoints in the corresponding groups (***P < 0.001, **P < 0.01, *P < 0.05) NS means there is no significant difference between the two groups, error line means ± SEM values.

After learning about the therapeutic effects of methimazole alone, we were eager to know how the addition of the potential prebiotic berberine would affect patients with GD. Six months after berberine combined with methimazole treatment, FT4, FT3 and TSH showed significant changes compared to baseline, with FT4, FT3 and TRAb showing a decreasing trend and TSH showing an increasing trend (**Figure 3**). In contrast to methimazole alone, berberine supplementation not only restored FT3 to the level (5.7 pmol/L) of healthy individuals but also restored TSH to a normal level (4.2 IU/mL). Moreover, the expression of TRAb at the end of treatment was much lower than that after treatment with methimazole alone, significantly improving the therapeutic effect on GD.

**Figure 4:**
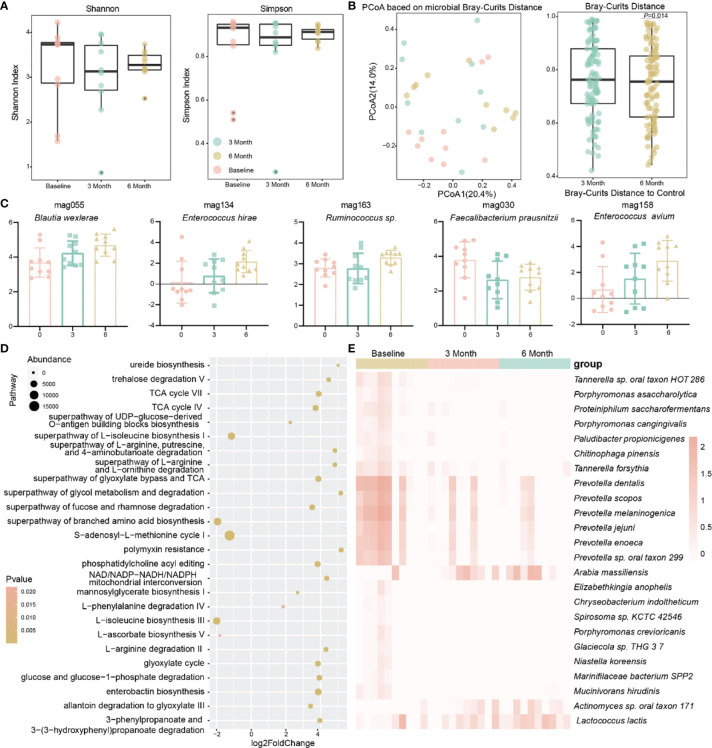
Effect of berberine combined with methimazole on the intestinal microbiota of patients with GD. **(A)** The effects of berberine combined with methimazole on intestinal microbiota α diversity, including shannon and Simpson index, the same color points represent the volunteers at different time points. **(B)** Principal coordinates analysis (PCoA) based on Bray-Curtis distances for macrogenomic species, with points of the same color representing the volunteer at different time points. **(C)** MAGs with significant differences from baseline at 6 months of treatment. **(D)** Gut microbial metabolic pathways that differ significantly from berberine combined with methimazole treatment at baseline for 6 months. **(E)** Strains with significant differences from baseline at 6 months of treatment.

In addition, the α diversity of the gut microbiota of the patients showed a tendency to decrease and then increase after methimazole treatment supplemented with berberine (**Figure 4A**). In terms of the microbiota structure, by calculating the Bray–Curtis distance, we found a significant change in the structure of the patients’ gut microbiota after 6 months compared to baseline (*P*=0.013) (**Figure 4B**), and the addition of berberine reshaped the structure of the patients’ gut microbiota.

Therefore, we further observed the changes in different bacteria in the intestines of the patients. In contrast to methimazole alone, the potential prebiotic berberine supplementation increased the abundance of beneficial bacteria such as *Lactococcus lactis* in the intestine and significantly decreased the abundance of pathogenic bacteria, including *Chryseobacterium indoltheticum* and *Tannerella forsythia*. In addition, it significantly reduced the abundance of *Prevotella* spp. (**Figure 4E**). Additionally, at the MAG level, the abundance of some potential probiotic bacteria, such as *Blautia wexlerae* and *Enterococcus hirae*, was significantly increased (**Figure 4C**). We observed a greater number of metabolic pathways responding to berberine supplementation compared to methimazole alone, with up to 117 metabolic pathways significantly altered compared to 8 with methimazole alone (**Figure 4D**). Among these differential metabolic pathways, enterobactin biosynthesis, ureide biosynthesis and L-phenylalanine degradation IV were significantly increased, and the superpathways of branched-chain amino acid biosynthesis and S-adenosyl-L-methionine cycle I were significantly decreased, implying that the addition of berberine increased the activity of the intestinal microbiota.

### The Potential Mechanism by Which Methimazole Combined With the Potential Prebiotic Berberine Improves the Therapeutic Effect on GD

**Figure 5:**
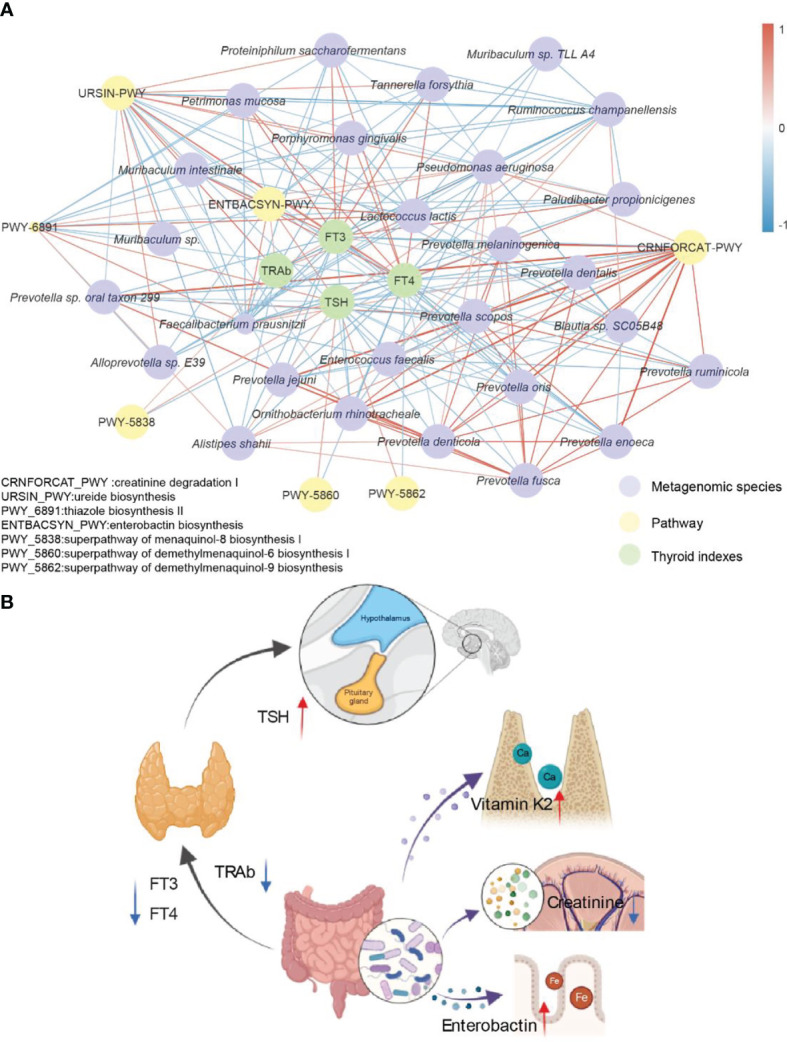
Potential mechanism of interaction between berberine combined with methimidazole and thyroid clinical indexes. **(A)** Significantly different strains, metabolic pathways and thyroid function indicators are based on Spearman correlation coefficients together to form this correlation network. Edge width and color (positive correlation in red, negative correlation in blue) are proportional to the correlation strength of the co-occurrence probabilities. Node size is proportional to the average abundance of the respective populations. **(B)** Simplified visualization schematic with possible mechanisms of action including the gut-hypothalamus axis and the gut-thyroid axis.

The combination of traditional Chinese and Western medicine could restore the indices of FT3 and TSH in patients with GD to the normal range, shift TRAb closer to the healthy level, and significantly improve the therapeutic effect on patients with GD. In addition, compared with the use of Western medicine alone, the combination of traditional Chinese and Western medicine significantly changed the intestinal microbiota of GD patients, increased the abundance of beneficial bacteria in GD patients, and decreased the abundance of pathogenic bacteria. To gain more insight into how the potential prebiotic berberine interacts with the gut microbiota to achieve improved efficacy, we then correlated the strains, the metabolic pathways that produced significant differences compared to baseline after 6 months of treatment and four thyroid indicators in the combined Chinese and Western medicine group (r > 0.4) (**Figure 5A**). After the addition of potential prebiotics berberine, *Faecalibacterium prausnitzii* and *Lactococcus lactis* were negatively correlated with FT3, FT4 and TRAb but positively correlated with TSH; *Faecalibacterium prausnitzii* was positively correlated with enterobacterin biosynthesis; and *Faecalibacterium prausnitzii* and *Lactococcus lactis* were positively correlated with the three metabolic pathways of vitamin K2 synthesis. FT4 was negatively correlated with *Faecalibacterium prausnitzii* and *Prevotella jejuni* and also negatively correlated with creatinine degradation I. We summarized a simple visualization mechanism diagram according to the relationships among the above indicators. These bacteria and their associated metabolic pathways use their downstream products to connect the gut, thyroid and hypothalamus, which in turn collectively affect the secretion of TSH, FT3, FT4 and TRAb (**Figure 5B**). This combined effect reshapes the patient’s thyroid function, relieves the condition and improves the outcome of the treatment.

## Discussion

We conducted a controlled clinical trial to determine the efficacy of methimazole and methimazole combined with the potential prebiotics berberine in the treatment of Graves’ disease. The results showed that methimazole supplemented with berberine was more effective than methimazole alone in treating GD and it significantly improved the thyroid function in GD patients. After 6 months of treatment, the FT3 of the two groups returned to the healthy level, but the addition of berberine also induced the TSH to reach a healthy state and significantly improved the patient’s condition. The TRAb index is an important index to reflect the recovery of thyroid patients (32). Although the two treatment methods reduced the content of TRAb in the patients, berberine intervention shifted the index closer to the healthy level. Therefore, the improvement of the therapeutic effect is closely related to berberine. In addition, Wang et al. used bicyclol combined with berberine in the treatment of hyperthyroidism, which is consistent with the results of this study (33). Previous studies have suggested that the pathogenesis of GD may be related to Th17 and Treg cells (Th17/Treg axis) (34, 35). MI treatment increases the percentage of Tregs in the body of adolescents with GD and promotes the normalization of thyroid hormone levels (36). Some articles have confirmed that berberine alleviates the disease by modulating the gut microbiota in mice with ulcerative colitis, thereby improving the Treg/Treg balance (37). During the treatment process, the positive superposition of the effects of Chinese medicine and Western medicine helps patients to better restore their thyroid function by regulating the immune system (38) of GD patients through adjusting their gut microbiota.

There has been much evidence showing that intestinal microbes may be another important cause of the development of thyroid disease in addition to genetic, autoimmune and other factors. The intestinal microbiota are related to the development of hyperthyroidism and they affect the indices of human thyroid function. In previous studies, it has been shown that the abundance of *Prevotella* spp. is higher in patients with GD than in the healthy population, and its altered abundance correlates with immune activity in humans, suggesting that the development of GD may be related to *Prevotella spp* (39). In our study, the abundance of *Prevotella* spp. was significantly reduced in both groups of patients after drug treatment and there was a reduction in the abundance of pathogenic bacteria such as *Streptococcus pneumoniae, Enterobacter hormaechei*, and *Chryseobacterium indologenes*. However, the addition of berberine significantly increased the abundance of beneficial bacteria such as *Lactococcus lactis* and *Enterococcus hirae* in patients compared to methimazole alone. Prebiotics are substances that reduce harmful strains of bacteria while beneficially promoting healthful strains or activities. In our study the addition of berberine increased the abundance of beneficial bacteria and showed prebiotic properties. Moreover, the increase in berberine also significantly modified the structure of the patients’ gut microbes. Compared to methimazole alone, the combination of berberine and methimazole intervention for 6 months significantly altered the Bray–Curtis distance compared to baseline (*P*=0.013).

These changes appear to be due to the action of the potential prebiotic berberine. It has been reported that berberine can alter the structure of the intestinal microbiota and reduce the α diversity (40), which is consistent with the results of the present study. In the combined Chinese and Western drugs group, FT3 and FT4 were negatively correlated with *Faecalibacterium prausnitzii*, TSH was positively correlated with *Faecalibacterium prausnitzii*, and *Lactococcus lactis* showed a strong negative correlation with FT4, indicating a close relationship between thyroid indicators and the microbiota.

*Faecalibacterium prausnitzii* and *Lactococcus lactis* play key roles in the whole network. AITD has been reported to be an organ-specific autoimmune disease mediated by T cells (41). However, it has been shown that *F. prausnitzii* can stimulate peripheral blood mononuclear cells and increase the secretion of the anti-inflammatory cytokine IL-10, which can suppress autoreactive T and B cells and maintain autoimmune tolerance (42–44). The microbiota plays a critical role in improving thyroid function in GD patients, and berberine adjusts the structure of the gut microbiota, which may produce beneficial metabolites to modulate host immunity and thus alleviate the disease. All of these results suggest that berberine combined with methimazole alleviates the symptoms of GD by modulating the gut microbiota.

In the network of correlations of FT3, FT4, TSH and TRAb with strains and metabolic pathways, we found some interesting phenomena. In our study, TSH was positively correlated with pathways such as *Faecalibacterium prausnitzii* and enterobactin biosynthesis, while a high positive correlation between *Faecalibacterium prausnitzii* and enterobactin biosynthesis was observed. In particular, berberine intervention resulted in a significant upregulation of enterobactin synthesis. Enterobactin is essential for iron uptake by the host as well as for the maintenance of homeostasis (45). In addition, iron(Fe) is one of the key micronutrients for maintaining thyroid function, and iron deficiency can cause thyroid function to decline (46). Therefore, upregulated enterobacterial synthesis may help to improve thyroid function in patients with GD. In conclusion, berberine combined with methimazole treatment may modulate the expression of metabolic pathways in the gut microbiota of hyperthyroid patients and contribute to the restoration of normal thyroid function in patients with GD.

Our study indicates that methimazole supplemented with berberine improves the therapeutic effect and restores thyroid function in patients with GD. In addition, the addition of the potential prebiotics berberine changed the gut microbiota structure of GD patients, increased the abundance of the beneficial bacterium *Lactococcus lactis*, decreased the abundance of the pathogenic bacterium *Chryseobacterium indologenes*, and regulated the microecological homeostasis of GD patients.

Moreover, this study reveals the potential mechanism by which berberine combined with methimazole may alleviate thyroid function by upregulating the synthesis of enterobactin and other pathways. Our study demonstrates the efficacy of adding the potential prebiotics berberine to the routine clinical treatment of GD and suggests the potential benefit of berberine combined with methimazole in modulating the composition of intestinal microbes during the treatment of GD, providing strong new evidence for the effectiveness of combining Chinese and Western drugs from the perspective of their effects on the intestinal microbiota.

## Data Availability Statement

The sequence data reported in this paper have been deposited in the NCBI database (resequencing and metagenomic sequencing data: PRJNA784925).

## Ethics Statement

The studies involving human participants were reviewed and approved by Hainan Provincial People’s Hospital. The patients/participants provided their written informed consent to participate in this study.

## Author Contributions

KC and DH designed the project. ZH, CC, and QO wrote the manuscript. YP and QO collected the samples and performed the experiments. ZH and JZ analyzed the data and generated the graphs. All authors read and approved the final paper.

## Funding

This research was supported by the Key Research and Development Project of Hainan Province (No. 321MS012 and No. ZDYF2018111).

## Conflict of Interest

The authors declare that the research was conducted in the absence of any commercial or financial relationships that could be construed as a potential conflict of interest.

## Publisher’s Note

All claims expressed in this article are solely those of the authors and do not necessarily represent those of their affiliated organizations, or those of the publisher, the editors and the reviewers. Any product that may be evaluated in this article, or claim that may be made by its manufacturer, is not guaranteed or endorsed by the publisher.

## References

[1] Frohlich E Wahl R. Microbiota and Thyroid Interaction in Health and Disease. Trends Endocrinol Metab (2019) 30(8):479–90. doi: 10.1016/j.tem.2019.05.008 31257166

[2] Zimmermann MB Boelaert K. Iodine Deficiency and Thyroid Disorders. Lancet Diabetes Endocrinol (2015) 3(4):286–95. doi: 10.1016/S2213-8587(14)70225-6 25591468

[3] Canaris GJ Manowitz NR Mayor G Ridgway EC. The Colorado Thyroid Disease Prevalence Study. Arch Intern Med (2000) 160(4):526–34. doi: 10.1001/archinte.160.4.526 10695693

[4] Nugent RA Jamison DT. What can a UN Health Summit do? Sci Transl Med (2011) 3(100):100cm25. doi: 10.1126/scitranslmed.3003132 21918103

[5] Taylor PN Albrecht D Scholz A Gutierrez-Buey G Lazarus JH Dayan CM. Global Epidemiology of Hyperthyroidism and Hypothyroidism. Nat Rev Endocrinol (2018) 14(5):301–16. doi: 10.1038/nrendo.2018.18 29569622

[6] Villanueva R Greenberg DA Davies TF Tomer Y. Sibling Recurrence Risk in Autoimmune Thyroid Disease. Thyroid (2003) 13(8):761–4. doi: 10.1089/105072503768499653 14558919

[7] Vaidya B Kendall-Taylor P Pearce SH. The Genetics of Autoimmune Thyroid Disease. J Clin Endocrinol Metab (2002) 87(12):5385–97. doi: 10.1210/jc.2002-020492 12466323

[8] De Leo S Lee SY Braverman LE. Hyperthyroidism. Lancet (2016) 388(10047):906–18. doi: 10.1016/S0140-6736(16)00278-6 PMC5014602 27038492

[9] Hai YP Lee ACH Frommer L Diana T Kahaly GJ. Immunohistochemical Analysis of Human Orbital Tissue in Graves' Orbitopathy. J Endocrinol Invest (2020) 43(2):123–37. doi: 10.1007/s40618-019-01116-4 31538314

[10] Sue M Akama T Kawashima A Nakamura H Hara T Tanigawa K. Propylthiouracil Increases Sodium/Iodide Symporter Gene Expression and Iodide Uptake in Rat Thyroid Cells in the Absence of TSH. Thyroid (2012) 22(8):844–52. doi: 10.1089/thy.2011.0290 PMC3407387 22853729

[11] Emiliano AB Governale L Parks M Cooper DS. Shifts in Propylthiouracil and Methimazole Prescribing Practices: Antithyroid Drug Use in the United States From 1991 to 2008. J Clin Endocrinol Metab (2010) 95(5):2227–33. doi: 10.1210/jc.2009-2752 PMC2869540 20335447

[12] Kalb RE Grossman ME. The Association of Aplasia Cutis Congenita With Therapy of Maternal Thyroid Disease. Pediatr Dermatol (1986) 3(4):327–30. doi: 10.1111/j.1525-1470.1986.tb00534.x 3774652

[13] Li X Liu GY Ma JL Zhou L. Risk of Congenital Anomalies Associated With Antithyroid Treatment During Pregnancy: A Meta-Analysis. Clinics (2015) 70(6):453–9. doi: 10.6061/clinics/2015(06)12 PMC4462563 26106966

[14] Mazhari A Emanuele MA Espiritu B. Desensitization to Methimazole. Endocr Pract (2021) 27(3):185–90. doi: 10.1016/j.eprac.2020.10.019 33779553

[15] Azizi F. The Safety and Efficacy of Antithyroid Drugs. Expert Opin Drug Saf (2006) 5(1):107–16. doi: 10.1517/14740338.5.1.107 16370960

[16] Tan S Chen L Jin L Fu X. The Efficiency and Safety of Methimazole and Propylthiouracil in Hyperthyroidism: A Meta-Analysis of Randomized Controlled Trials. Medicine (Baltimore) (2021) 100(30):e26707. doi: 10.1097/MD.0000000000026707 34397700 PMC8322508

[17] Hu XY Zhang H Rong YY Zhang MH Zhang XN. [Clinical Observation on Treatment of Type 2 Cardiac and Kidney Syndrome by Combination of Traditional Chinese and Western Medicines]. Zhongguo Zhong Yao Za Zhi (2017) 42(19):3815–8. doi: 10.19540/j.cnki.cjcmm.20170901.009 29235300

[18] Riva DA Fernandez-Larrosa PN Dolcini GL Martinez-Peralta LA Coulombie FC Mersich SE. Two Immunomodulators, Curcumin and Sulfasalazine, Enhance IDV Antiretroviral Activity in HIV-1 Persistently Infected Cells. Arch Virol (2008) 153(3):561–5. doi: 10.1007/s00705-007-0023-4 18175040

[19] Ren J Fu L Nile SH Zhang J Kai G. Salvia Miltiorrhiza in Treating Cardiovascular Diseases: A Review on Its Pharmacological and Clinical Applications. Front Pharmacol (2019) 10:753. doi: 10.3389/fphar.2019.00753 31338034 PMC6626924

[20] Hu Y Zhou X Liu P Wang B Duan DM Guo DH. A Comparison Study of Metformin Only Therapy and Metformin Combined With Chinese Medicine Jianyutangkang Therapy in Patients With Type 2 Diabetes: A Randomized Placebo-Controlled Double-Blind Study. Complement Ther Med (2016) 24:13–8. doi: 10.1016/j.ctim.2015.11.005 26860796

[21] Liu D Liang XC. New Developments in the Pharmacodynamics and Pharmacokinetics of Combination of Chinese Medicine and Western Medicine. Chin J Integr Med (2017) 23(4):312–9. doi: 10.1007/s11655-016-2271-1 27921195

[22] Hua M Liu Z Sha J Li S Dong L Sun Y. Effects of Ginseng Soluble Dietary Fiber on Serum Antioxidant Status, Immune Factor Levels and Cecal Health in Healthy Rats. Food Chem (2021) 365:130641. doi: 10.1016/j.foodchem.2021.130641 34325349

[23] Xiao Z Deng Q Zhou W Zhang Y. Immune Activities of Polysaccharides Isolated From Lycium Barbarum L. What do We Know So Far? Pharmacol Ther (2021) 107921:16. doi: 10.1016/j.pharmthera.2021.107921 34174277

[24] Li M Shu X Xu H Zhang C Yang L Zhang L. Integrative Analysis of Metabolome and Gut Microbiota in Diet-Induced Hyperlipidemic Rats Treated With Berberine Compounds. J Transl Med (2016) 14(1):237. doi: 10.1186/s12967-016-0987-5 27495782 PMC4975912

[25] Xu X Gao Z Yang F Yang Y Chen L Han L. Antidiabetic Effects of Gegen Qinlian Decoction *via* the Gut Microbiota Are Attributable to Its Key Ingredient Berberine. Genomics Proteomics Bioinformatics (2020) 18(6):721–36. doi: 10.1016/j.gpb.2019.09.007 PMC8377040 33359679

[26] Li D Liu CM Luo R Sadakane K Lam TW. MEGAHIT: An Ultra-Fast Single-Node Solution for Large and Complex Metagenomics Assembly *via* Succinct De Bruijn Graph. Bioinformatics (2015) 31(10):1674–6. doi: 10.1093/bioinformatics/btv033 25609793

[27] Wood DE Salzberg SL. Kraken: Ultrafast Metagenomic Sequence Classification Using Exact Alignments. Genome Biol (2014) 15(3):R46. doi: 10.1186/gb-2014-15-3-r46 24580807 PMC4053813

[28] Franzosa EA McIver LJ Rahnavard G Thompson LR Schirmer M Weingart G. Species-Level Functional Profiling of Metagenomes and Metatranscriptomes. Nat Methods (2018) 15(11):962–8. doi: 10.1038/s41592-018-0176-y PMC6235447 30377376

[29] Kang DD Froula J Egan R Wang Z. MetaBAT, an Efficient Tool for Accurately Reconstructing Single Genomes From Complex Microbial Communities. PeerJ (2015) 3:e1165. doi: 10.7717/peerj.1165 26336640 PMC4556158

[30] Chaumeil PA Mussig AJ Hugenholtz P Parks DH. GTDB-Tk: A Toolkit to Classify Genomes With the Genome Taxonomy Database. Bioinformatics (2019) 36:1925–7. doi: 10.1093/bioinformatics/btz848 PMC7703759 31730192

[31] Huo D Cen C Chang H Ou Q Jiang S Pan Y. Probiotic Bifidobacterium Longum Supplied With Methimazole Improved the Thyroid Function of Graves' Disease Patients Through the Gut-Thyroid Axis. Commun Biol (2021) 4(1):1046. doi: 10.1038/s42003-021-02587-z 34493790 PMC8423791

[32] Barbesino G Tomer Y. Clinical Review: Clinical Utility of TSH Receptor Antibodies. J Clin Endocrinol Metab (2013) 98(6):2247–55. doi: 10.1210/jc.2012-4309 PMC3667257 23539719

[33] Wang Y. Efficacy of Bicyclol Combined With Berberine on Hyperthyroidism Patients and Analysis of Related Factors Inducing the Disease. Int J Clin Exp Med (2020) 13(5):3027–34.

[34] Papamichael KX Papaioannou G Karga H Roussos A Mantzaris GJ. Helicobacter Pylori Infection and Endocrine Disorders: Is There a Link? World J Gastroenterol (2009) 15(22):2701–7. doi: 10.3748/wjg.15.2701 PMC2695884 19522019

[35] Bassi V Santinelli C Iengo A Romano C. Identification of a Correlation Between Helicobacter Pylori Infection and Graves' Disease. Helicobacter (2010) 15(6):558–62. doi: 10.1111/j.1523-5378.2010.00802.x 21073613

[36] Klatka M Grywalska E Partyka M Charytanowicz M Kiszczak-Bochynska E Rolinski J. Th17 and Treg Cells in Adolescents With Graves' Disease. Impact of Treatment With Methimazole on These Cell Subsets. Autoimmunity (2014) 47(3):201–11. doi: 10.3109/08916934.2013.879862 24443787

[37] Cui H Cai Y Wang L Jia B Li J Zhao S. Berberine Regulates Treg/Th17 Balance to Treat Ulcerative Colitis Through Modulating the Gut Microbiota in the Colon. Front Pharmacol (2018) 9:571. doi: 10.3389/fphar.2018.00571 29904348 PMC5991375

[38] Li S Wang N Tan HY Chueng F Zhang ZJ Yuen MF. Modulation of Gut Microbiota Mediates Berberine-Induced Expansion of Immuno-Suppressive Cells to Against Alcoholic Liver Disease. Clin Transl Med (2020) 10(4):e112. doi: 10.1002/ctm2.112 32790968 PMC7438809

[39] Bongers G Pacer ME Geraldino TH Chen L He Z Hashimoto D. Interplay of Host Microbiota, Genetic Perturbations, and Inflammation Promotes Local Development of Intestinal Neoplasms in Mice. J Exp Med (2014) 211(3):457–72. doi: 10.1084/jem.20131587 PMC3949565 24590763

[40] Zhang JD Liu J Zhu SW Fang Y Wang B Jia Q. Berberine Alleviates Visceral Hypersensitivity in Rats by Altering Gut Microbiome and Suppressing Spinal Microglial Activation. Acta Pharmacol Sin (2021) 0:1–13. doi: 10.1038/s41401-020-00601-4 PMC8563748 33558654

[41] Antonelli A Ferrari SM Corrado A Di Domenicantonio A Fallahi P. Autoimmune Thyroid Disorders. Autoimmun Rev (2015) 14(2):174–80. doi: 10.1016/j.autrev.2014.10.016 25461470

[42] Sokol H Pigneur B Watterlot L Lakhdari O Bermudez-Humaran LG Gratadoux JJ. Faecalibacterium Prausnitzii is an Anti-Inflammatory Commensal Bacterium Identified by Gut Microbiota Analysis of Crohn Disease Patients. Proc Natl Acad Sci USA (2008) 105(43):16731–6. doi: 10.1073/pnas.0804812105 PMC2575488 18936492

[43] Kristensen B. Regulatory B and T Cell Responses in Patients With Autoimmune Thyroid Disease and Healthy Controls. Dan Med J (2016) 63(2):B5177. 26836805

[44] Pyzik A Grywalska E Matyjaszek-Matuszek B Rolinski J. Immune Disorders in Hashimoto's Thyroiditis: What Do We Know So Far? J Immunol Res (2015) 2015:8. doi: 10.1155/2015/979167 PMC4426893 26000316

[45] Qi B Han M. Microbial Siderophore Enterobactin Promotes Mitochondrial Iron Uptake and Development of the Host *via* Interaction With ATP Synthase. Cell (2018) 175(2):571–82.e11. doi: 10.1016/j.cell.2018.07.032 30146159

[46] Beck GE. Blood Iron Level in Relation to Thyroid Function. Clin Lat (1953) 3(2):56–76. 13106956

